# CEUS LI-RADS for diagnosis of hepatocellular carcinoma in individuals without LI-RADS-defined hepatocellular carcinoma risk factors

**DOI:** 10.1186/s40644-023-00541-2

**Published:** 2023-03-06

**Authors:** Zhe Huang, Ping Ping Zhou, Shan Shan Li, Kaiyan Li

**Affiliations:** Department of Medical Ultrasound, Tongji Hospital, Tongji Medical College, Huazhong University of Science and Technology, 1095 Jiefang Avenue, Postal address 430030, Qiaokou District, Wuhan, Hubei Province China

**Keywords:** Contrast-Enhanced Ultrasound, Hepatocellular carcinoma, Risk factors, Diagnosis

## Abstract

**Purpose:**

This study evaluated the performance of the contrast-enhanced ultrasound (CEUS) Liver Imaging Reporting and Data System (LI-RADS) in patients without LI-RADS-defined hepatocellular carcinoma (HCC) risk factors (RF−).

**Methods:**

Patients with LI-RADS-defined HCC risk factors (RF+) and RF− were enrolled in a retrospective study. Additionally, a prospective evaluation in the same centre was performed as a validation set. The diagnostic performances of the CEUS LI-RADS criteria in RF+ and RF− patients were compared.

**Results:**

Overall, we included 873 patients in the analyses. In the retrospective study, the LI-RADS category (LR)-5 specificities for diagnosing HCC did not differ between the RF+ and RF− groups (77.5% [158/204] vs 91.6% [196/214], *P* = 0.369, respectively). However, the positive predictive value (PPV) of CEUS LR-5 was 95.9% (162/169) and 89.8% (158/176) in the RF+ and RF− groups, respectively (*P* = 0.029). In the prospective study, the PPV of LR-5 for HCC lesions was significantly higher in the RF+ group than in the RF− group (*P* = 0.030). The sensitivity and specificity did not differ between the RF+ and RF− groups (*P* = 0.845 and *P* = 0.577, respectively).

**Conclusions:**

The CEUS LR-5 criteria shows clinical value for diagnosis of HCC in patients with and without risks.

## Introduction

Hepatocellular carcinoma (HCC) is among the top five leading causes of cancer-related mortality worldwide [1]. To standardise the interpretation and reporting of HCC, the American College of Radiology developed the Liver Imaging Reporting and Data System (LI-RADS) to reduce variability in imaging reports and improve communication among clinicians. LI-RADS provides a comprehensive algorithm, including major features (e.g. lesion size, enhancement pattern, timing, and degree of washout) and ancillary features that can be used to classify liver observations based on their likelihood of being HCC, ranging from definitively benign (LI-RADS category 1 [LR-1]) to definitively HCC (LR-5).

Previous studies have confirmed that the standard contrast-enhanced ultrasound (CEUS) LI-RADS classification has high clinical value for diagnosing HCC [2, 3]. However, the CEUS LI-RADS was introduced specifically for patients at risk of HCC (i.e. patients with liver cirrhosis, chronic hepatitis B, or a history of HCC). CEUS LI-RADS studies have mainly focused on patients with LI-RADS-defined risk factors for HCC (RF+), specifically patients with hepatitis B virus (HBV) infection [4, 5]. Liver cirrhosis, mainly caused by HBV infection, is the main risk factor for HCC in China and some sub-Saharan countries, such as Nigeria, Namibia, Gabon, Cameroon, and Burkina Faso [6, 7]. Approximately 54% of HCC cases worldwide can be attributed to HBV infection, affecting 400 million people globally [8]. However, governmental promotion of hepatitis B vaccinations in numerous regions has reduced the incidence of HBV infection and HBV-related liver cancer [9].

Hepatitis C virus infection is currently a prominent global health problem, and the incidence of hepatitis C infections has increased in both Western countries and China in recent years [10]. Furthermore, non-alcoholic fatty liver disease (NAFLD) has become a major cause of HCC owing to the current increase in the prevalence of obesity and diabetes. The incidence of NAFLD-related HCC has risen steadily, with ~ 20% of HCC cases attributable to NAFLD [11]. However, such patients do not classify as RF+. As the proportion of such patients increases, the diagnostic performance of the CEUS LI-RADS in patients with no LI-RADS-defined risk factors for HCC (RF−) requires further investigation.

Radical surgical resection, locoregional approaches (e.g. radiofrequency ablation and chemoembolisation), and liver transplantation represent potentially curative treatment modalities for patients with early-stage Barcelona Clinic Liver Cancer 0/A (BCLC 0/A) and even intermediate-stage (BCLC B) HCC [12]; HCC patients with BCLC C/D were considered to have a poor prognosis [13]. Emerging investigational immune-based combinations might add to the current treatment landscape. Following the results of the IMbrave150 trial, atezolizumab plus bevacizumab has become the new first-line treatment for advanced HCC [14]. However, several other immune-based combinations are also under assessment [15]. For instance, metronomic capecitabine is a potential therapeutic alternative for patients with Child-Pugh B scores who cannot be prescribed sorafenib due to regulatory restrictions and intolerance to tyrosine-kinase inhibitors [16]. Thus, it is meaningful to expand the application range of CEUS LI-RADS in this setting.

Consequently, this study evaluated the performance of the CEUS LI-RADS criteria in RF− patients.

## Methods

### Patient selection

Our institutional review board approved these retrospective and prospective studies. All patients participating in the clinical trials provided written informed consent. A retrospective evaluation of continuous liver CEUS images was performed between January 2013 and January 2021. A prospective evaluation was performed in the same centre between February 2021 and August 2021 as a validation set.

**Fig. 1:**
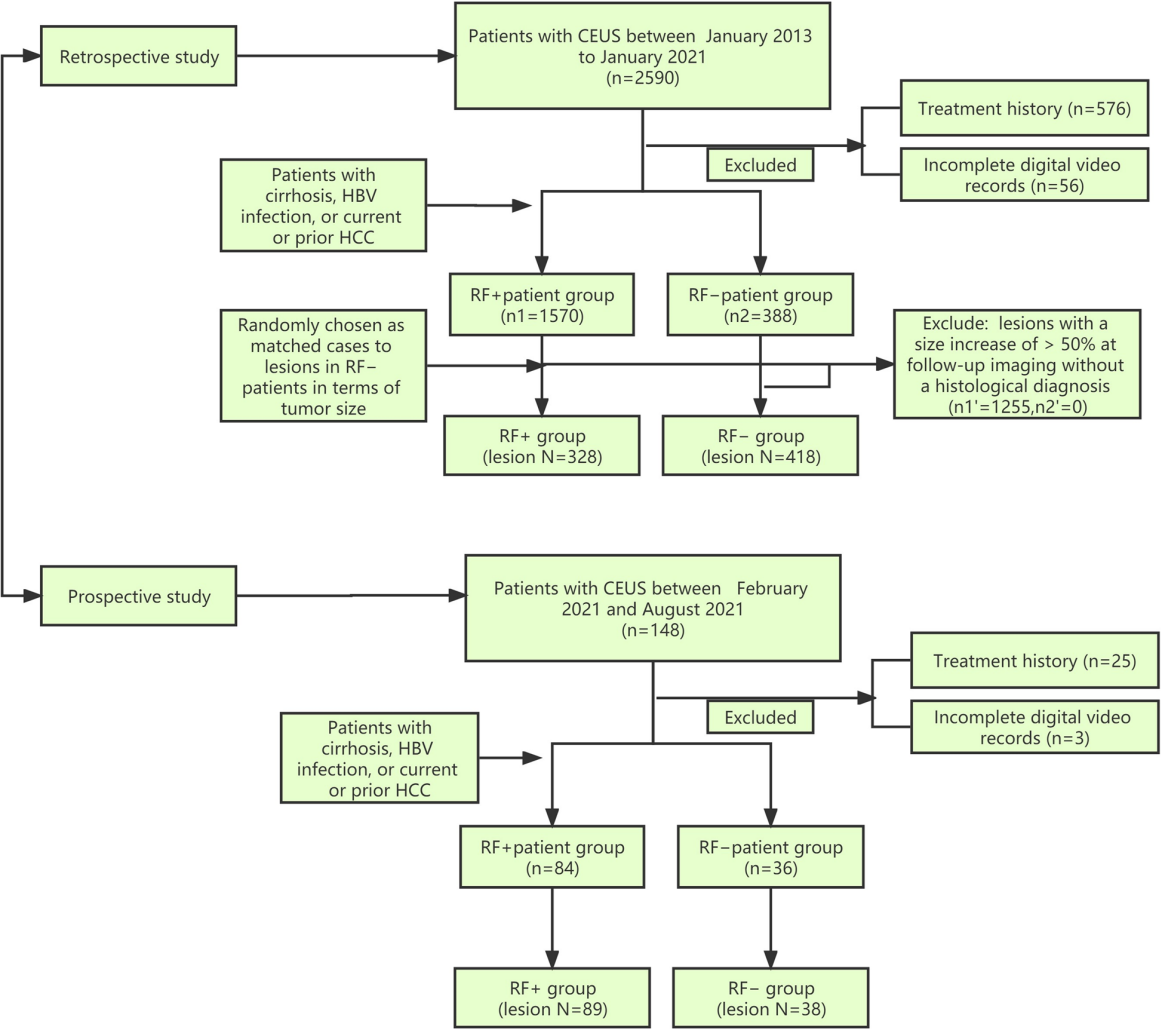
Risk factor stratification algorithm. For patients without laboratory evidence of chronic HBV infection, clinical interpretations of non-tumour liver pathology specimens obtained within one year of the imaging study selected for LI-RADS assessment were used (when available) to stratify patients into RF+ and RF− groups. For patients without non-tumour liver pathology specimens, the imaging study selected for LI-RADS assessment was reviewed by an author not involved in the LI-RADS interpretation who looked for evidence of gross surface nodularity (i.e. definite cirrhosis by imaging). For patients without definite cirrhosis by imaging, an FIB-4 index calculation, a validated tool for the non-invasive prediction of advanced fibrosis (i.e. cirrhosis), was attempted [17]. Patients without the laboratory values necessary for the FIB-4 calculation within 30 days of the LI-RADS imaging study or with a FIB-4 in the 1.45–3.25 range were considered to have an indeterminate risk status. These cases were excluded. ALT = alanine transaminase; AST = aspartate transaminase; HBV = hepatitis B virus; LI-RADS = Liver Imaging Reporting and Data System; RF− = not high-risk for hepatocellular carcinoma per LI-RADS criteria; RF+ = high-risk for hepatocellular carcinoma per LI-RADS criteria; FIB-4 = Fibrosis 4 Score

The inclusion criteria were: (1) the presence of untreated liver lesions at the time of the initial CEUS examination; (2) the availability of complete electronic medical records, CEUS data for the lesion, and digital video records; and (3) visibility of all lesions during the baseline ultrasound examination. A total of 418 lesions in RF− patients from January 2013 to January 2021 were included in the retrospective study. Three-hundred-twenty-eight lesions in RF+ patients from the same period were randomly chosen as matched cases to the lesions (in terms of tumour size) in the RF− patients (Fig. 1). A total of 38 lesions in 36 RF− patients and 89 lesions in 84 RF+ patients from February 2021 and August 2021 were included in the prospective study (Fig. 1). All patients underwent CEUS prior to surgery and received no previous treatments, such as transarterial chemoembolisation, chemotherapy, and radiotherapy.

**Fig. 2:**
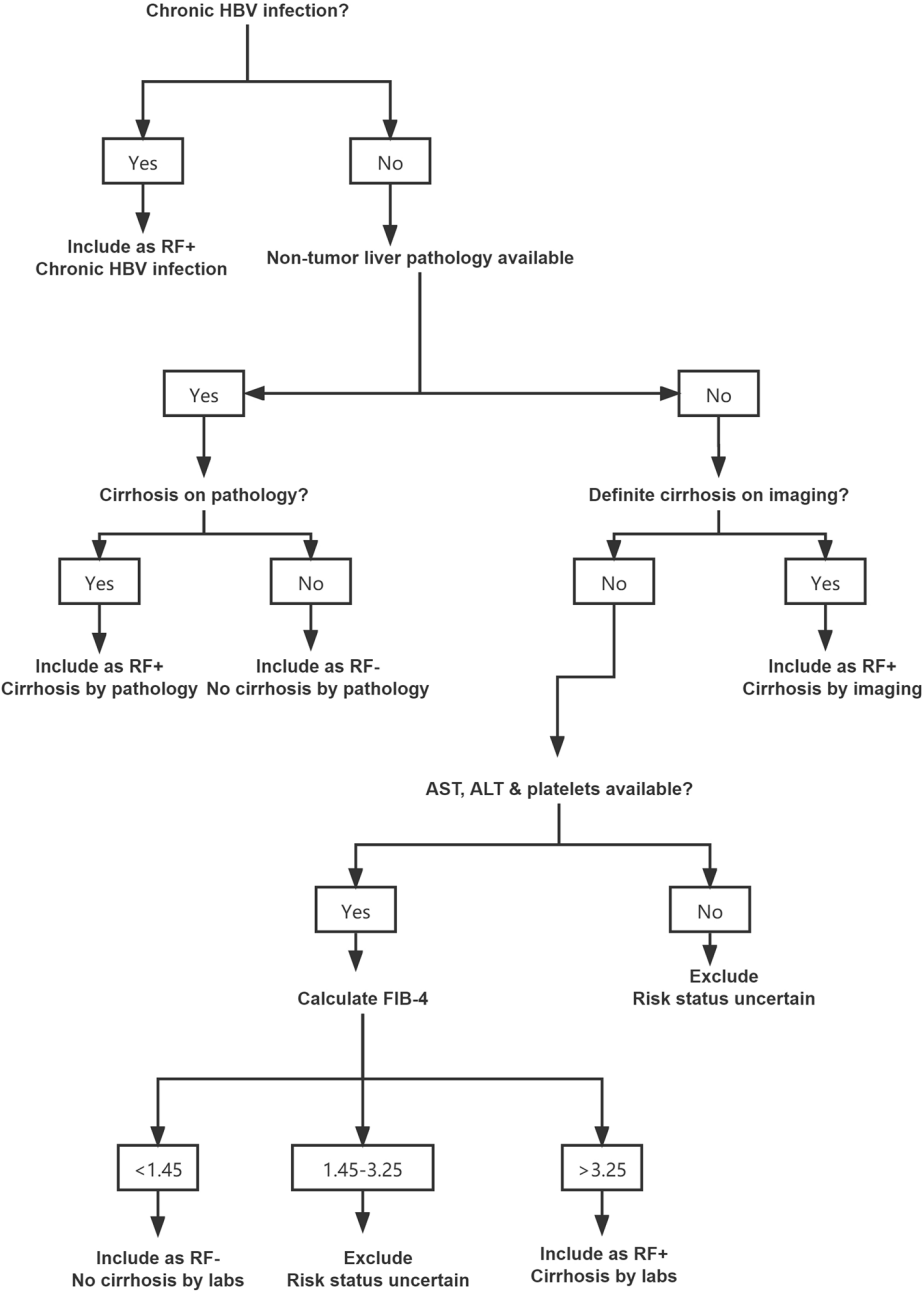
Flow diagram for RF+ and RF− groups. ALT = alanine transaminase; AST = aspartate transaminase; HBV = hepatitis B virus; LI-RADS = Liver Imaging Reporting and Data System; RF− = not high-risk for hepatocellular carcinoma per LI-RADS criteria; RF+ = high-risk for hepatocellular carcinoma per LI-RADS criteria; FIB-4 = Fibrosis 4 Score

RF+ patients were those with cirrhosis, HBV infection, or current or prior HCC. RF− patients were those with none of the above risk factors [18]. A risk factor assessment algorithm (Fig. 2) was devised to classify the patients as RF+ or RF− using the CEUS LI-RADS criteria. This algorithm utilises a hierarchy of laboratory, imaging, and pathology data designed to determine the risk status with the highest possible certainty.

### Ultrasound examination

CEUS was performed using the GE Logiq 9 device (GE Healthcare, Wauwatosa, WI, USA) with a 3–5 L probe and EPIQ 7 device (Philips Medical Solutions, Mountain View, CA, USA) with a C5–1 probe. Each patient underwent comprehensive conventional grayscale liver ultrasonography prior to CEUS. The number, location, size, shape and blood flow distribution of intrahepatic nodules were recorded. Instruct the patient to breathe calmly, select the best section to display the nodule, fix the probe position, and switch to CEUS mode. Thereafter, 2.4 mL of a contrast agent (SonoVue, Bracco, Milan, Italy) was injected intravenousl, followed by a bolus of 10 mL 0.9% saline. At the same time sonographers start the timer. A mechanical index of 0.08 was used for CEUS. The region of interest was imaged until liver parenchymal enhancement faded, typically after 3 min or longer. The CEUS examinations were recorded as video clips for analysis. Acquisition protocols have remained the same from 2013 until 2021. Two sonographers (with more than 10 years and 18 years of experience with CEUS, respectively), who were blinded to the patients’ histopathology results performed CEUS.

### Reference standard

All malignant lesions, including both HCC and non-HCC malignancies, were diagnosed based on the findings of histopathologic examinations. The reference standard for benign lesions was either histopathologic assessment or follow-up CT or MRI imaging. In patients whose lesions were classified as CEUS LR-1, contrast-enhanced CT or MRI was used as the reference standard. For patients whose lesions were classified as CEUS LR-2, LR-3, and LR-4, follow-up imaging or tissue sampling was used as the reference standard. If the lesion increased by < 50% within 12 months and did not progress to a higher CEUS LI-RADS category in subsequent imaging examinations, it was classified as benign. Observations for lesions with a size increase of > 50% at follow-up imaging without a histological diagnosis were removed from the analysis because of the lack of a reference standard.

### Contrast-enhanced ultrasound imaging analysis

Two sonographers (with 8 years and 5 years of experience with CEUS, respectively), who were blinded to the patients’ histopathology results, assigned categories according to CEUS LI-RADS (2017 edition). A radiologist evaluated each lesion in patients with multiple lesions. Before image analysis, each radiologist attended a 1-h lecture on the details of CEUS LI-RADS (2017 edition) and a hands-on instruction session with 20 practical cases selected from the excluded population. They had completed standardized training for resident doctors. The training period involved both US examinations in daily clinical practice. If the two radiologists’ opinions differed, another radiologist (blinded, with more than 20 years of experience) arbitrated the final decision. A full description can be found on the official website of the American.

College of Radiology. APHE is defined as entirely or partially (neither rimlike nor peripheral discontinuous) hyperechoic compared with the surrounding parenchyma. Washout is defined as whole or partial hypoen-hancement relative to liver beginning in or after the arterial phase. Early washout occurs within 60 seconds after injection of the contrast agent. Punched-out appearance is defined when the nodule becomes markedly hypoenhanced (appears black). Marked washout is assigned when punched-out appearance occurs within 2 minutes (otherwise defined as mild).

### Statistical analyses

Continuous variables were expressed as the means ± standard deviations. Qualitative data were presented as numbers and percentages. Differences between groups were analysed using Wilcoxon rank-sum test, Pearson’s chi-squared test, or Fisher’s exact test, as appropriate. The estimated values of sensitivity, specificity, positive predictive value (PPV), negative predictive value, and accuracy of the CEUS LI-RADS in the RF+ group and RF− group, for the diagnosis of HCC, were compared using the McNemar test. Inter-observer agreement was assessed using the k statistic: 0–0.20, poor agreement; 0.21–0.40, fair agreement; 0.41–0.60, moderate agreement; 0.61–0.80, approximately equal agreement; and 0.81–1.00, excellent agreement. *P*-values < 0.05 were considered statistically significant. All statistical analyses were performed using SPSS software (Version 23.0, IBM, Armonk, NY, USA).

## Results

### Patient and liver nodule characteristics in the retrospective study

The RF− group included 418 lesions from 388 patients (Fig. 2). Among these, three lesions each were observed in two patients, and two lesions each in 26 patients. The mean nodule diameter was 36.5 ± 21.7 (7–196) mm. The RF+ group included 328 lesions from 315 patients, with two lesions each in 13 patients; the mean lesion diameter was 33.4 ± 21.6 (8–131) mm. Table 1 presents the clinical characteristics of the patients, including age, sex, and tumour histopathology. The time interval between the CEUS and surgery was 7 ± 4 days.

**Table 1:** Clinical and pathologic information

Characteristic	RF+ group (*n* = 315)	RF− group (*n* = 388)	*P*
Mean age (y)^a^	52.4 ± 12.8 (22–75)	49.7 ± 12.0 (17–86)	0.061
Sex			
Men	265 (76.7)	306 (71.5)	0.076
Mean nodule size (mm)^a^	33.4 ± 21.6 (8–131)	36.5 ± 21.7 (7–196)	0.391
Liver backgrounds			
HBV infection	243 (77.1)		
Cirrhosis	218 (69.2)		
NAFLD- non cirrhosis		123 (31.7)	
HCV infection- non cirrhosis		198 (51.0)	
Other liver diseases- non cirrhosis		67 (17.3)	
Liver nodules	(w = 328)	(w = 418)	
Pathologic Analysis			
HCC	206 (62.8)	204 (48.8)	< 0.001
DN/RN	6 (1.8)	7 (1.7)	0.873
Focal nodular hyperplasia	13 (4.0)	28 (6.7)	0.104
Hemangioma	15 (4.6)	25 (6.0)	0.397
ICC	18 (5.5)	38 (9.1)	0.064
HCC-ICC	4 (1.2)	2 (0.5)	0.261
Biliary adenoma	3 (0.9)	4 (1.0)	0.953
Neuroendocrine neoplasm	8 (2.4)	12 (2.9)	0.717
Lymphoma	1 (0.3)	1 (0.2)	0.863
Angiomyolipoma.	2 (0.6)	8 (1.9)	0.124
Inflammatory pseudotumor	17 (5.2)	32 (7.7)	0.176
Lipoma	2 (0.6)	7 (1.7)	0.201
No pathologic analysis			
Follow-up CT or MRI imaging - Hemangioma	34 (10.4)	50 (12.0)	0.494

*Note*: Unless otherwise indicated, data are liver nodules(w = 328 or 418) or patients (n = 315 or 388) and data in parentheses are percentages. Mean data are ± standard deviation. *HBV* Hepatitis B Virus, *HCV* Hepatitis C Virus, *NAFLD* non-alcoholic fatty liver disease, *NA* not available, *HCC* hepatocellular carcinoma, *DN* dysplastic nodule, *RN* regenerative nodule, *ICC* inrrahepatic cholangiocarcinoma

^a^ Data in parentheses are range

### Category distributions in the RF− group in the retrospective study

Table 2 lists the ultrasound characteristics of the 418 liver lesions in the RF− group. Neither rim-like nor peripheral discontinuous hyper-enhancement was observed in 191 lesions, including 184 lesions with homogeneous hyper-enhancement, two lesions with heterogenous hyper-enhancement, and five lesions with rim hyper-enhancement.

**Table 2:** Imaging characteristics of different typos of liver nodules in rf- patients in retrospective study

Image Features	Malignant Lesions (*n* = 245)		Benign Lesions (*n* = 173)								Total
	HCC (*n* = 204)	OM (*n* = 41)	DN/RN (*n* = 22)	FNH (*n* = 28)	Hemangiom (*n* = 60)	Biliary adenoma (*n* = 4)	AML (*n* = 8)	IPT (*n* = 32)	Lipoma (*n* = 7)	NEN (*n* = 12)	
Gray-scale echogenicity											
Hyperechoic	20	3	1	2	57	0	5	2	7	9	106
Hypoechoic	184	38	21	26	3	4	3	30	0	3	312
Arterial phase											
Hyperenhancemenr											
Homogeneous	184	6	18	28	31	2	7	29	3	11	319
Inhomogenous	2	2	0	0	14	0	0	0	0	1	19
Rim	5	20	0	0	2	0	1	1	0	0	29
Peripheral nodular	7	13	0	0	13	0	0	0	0	0	33
Isoenhancement	3	0	4	0	0	2	0	2	4	0	15
Hypoenhancement	3	0	0	0	0	0	0	0	0	0	3
Late phase enhancements											
Isoenhancement	28	0	13	19	35	4	3	18	3	5	128
Hypoenhancement	172	41	4	0	7	0	2	12	0	2	240
Hyperenhancement	4	0	5	9	18	0	3	2	4	5	50
Washout											
No washout	32	0	18	28	53	4	6	20	7	10	178
delayed and moderate	158	2	4	0	7	0	2	12	0	2	187
< 60 s	9	22	0	0	0	0	0	1	0	0	32
Marked, ≤120 s	2	18	0	0	0	0	0	0	0	0	20

*Note*: Data are numbers of nodules. *HCC* hepatocellular carcinoma, *OM* Other malignant nodules (including lymphoma, inrrahepatic cholangiocarcinoma and HCC-ICC), *DN* dysplastic nodule, *RN* regenerative nodule, *AML* angiomyolipoma, *IPT* inflammatory pseudotumor, *NEN* neuroendocrine neoplasm

A full description can be found on the official website of the American College of Radiology (https://www.acr.org/Clinical-Resources/Reporting-and-Data-Systems/LI-RADS/CEUS-LI-RADS-v2017). Washout is defined as whole or partial hypoenhancement relative to liver beginning in or after the arterial phase. Early washout occurs within 60 seconds after injection of the contrast agent. Punched-out appearance is defined when the nodule becomes markedly hypoenhanced (appears black). Marked washout is assigned when punched-out appearance occurs within 2 minutes (otherwise defined as mild)

**Fig. 3:**
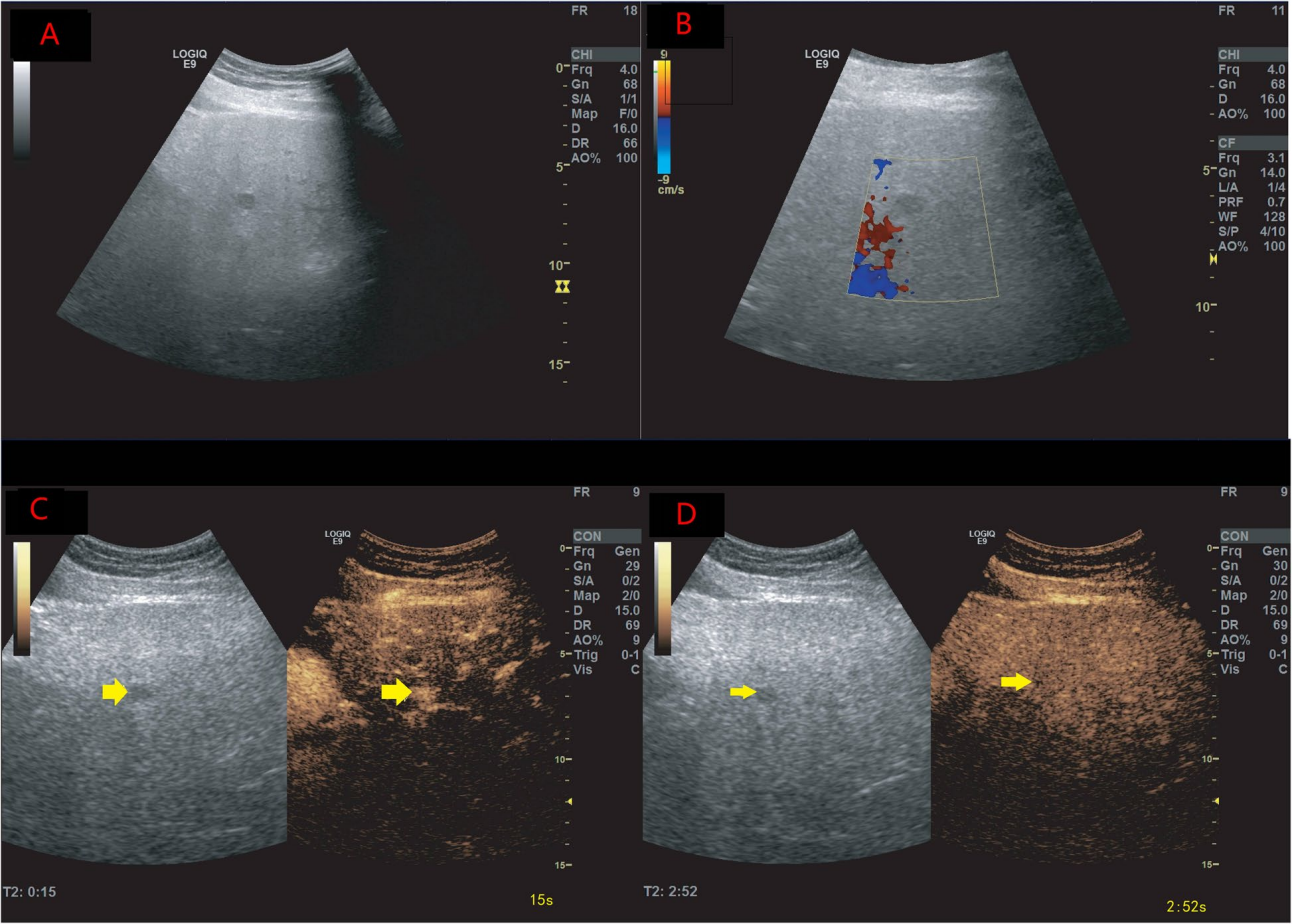
A liver mass was discovered during ultrasound examination in a 52-year-old woman with non-alcoholic fatty liver disease and without risk for HCC. The focal liver lesions were estimated to be about 1.3 cm in diameter and located in the right lobes (**A**). Colour Doppler flow imaging revealed no obvious blood flow in the lesions (**B**). On CEUS, the mass displayed hyper-enhancement after the injection of contrast agent (**C**) and no washout (**D**). The lesion was classified as LR-4 according to the CEUS LI-RADS guidelines. Histopathology revealed the lesion to be an angiomyolipoma. CEUS = contrast-enhanced ultrasound; HCC = hepatocellular carcinoma; LI-RADS = Liver Imaging Reporting and Data System; LR = LI-RADS category

**Fig. 4:**
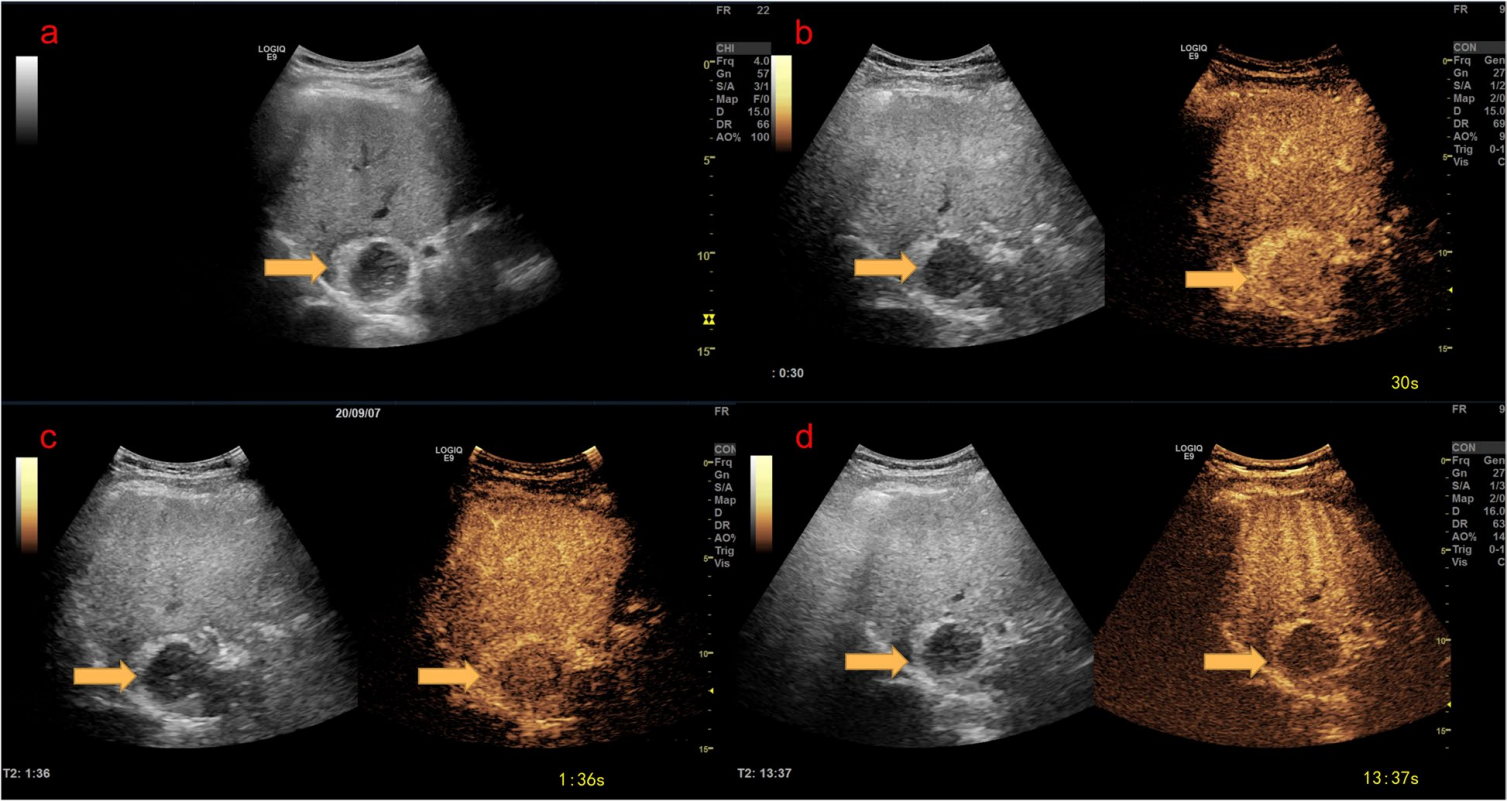
A liver mass discovered during physical examination in a 30-year-old male RF– patient. The mass was estimated to measure approximately 4.6 × 3.6 cm. A hypoechoic mass located in the caudate lobe with peripheral hyperechoic rim (fat) (**A**). On CEUS, the mass displayed hyperenhancement after contrast agent injection (**B**). Contrast agent washout was observed as a hypoenhancement at the lesion during the portal venous phase (156 s) (**C**) and hypoenhancing during the late phase (13 min 37 s) (**D**). The ‘Arterial phase hyperenhancement with whole showing marked washout in late in onset (60 s)’ lesion was classified as LR-5 based on the CEUS LI-RADS guidelines. Histopathology revealed the lesion to be an angiomyolipoma. CEUS = contrast-enhanced ultrasound; LI-RADS = Liver Imaging Reporting and Data System; LR = LI-RADS category; RF− = not high-risk for hepatocellular carcinoma per LI-RADS criteria

**Fig. 5:**
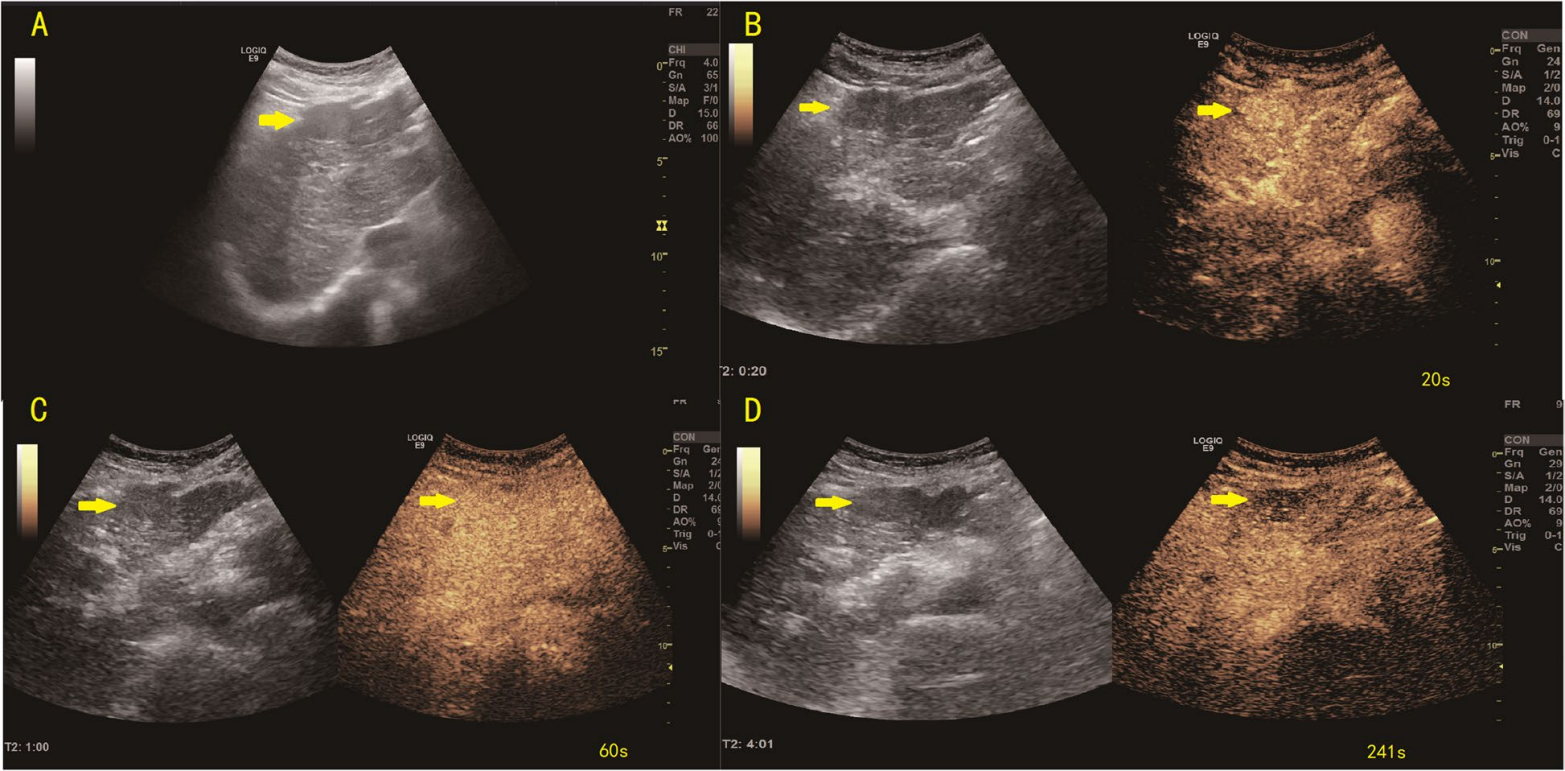
Typical HCC CEUS imaging. The mass is estimated to measure approximately 2.8 × 2.2 cm. A hypoechoic mass located in the left lobe (**A**). On CEUS, the mass displayed hyperenhancement after contrast agent injection (**B**). Contrast agent washout was observed as a isoenhancement at the lesion during the portal venous phase (60 s) (**C**) and hypoenhancing during the late phase (4 min 01 s) (**D**). The ‘Arterial phase hyperenhancement with whole showing marked washout in late in onset (60 s)’ lesion was classified as LR-5 based on the CEUS LI-RADS guidelines. CEUS = contrast-enhanced ultrasound; HCC = hepatocellular carcinoma; LI-RADS = Liver Imaging Reporting and Data System; LR = LI-RADS category

Washout was observed in 239 of 418 liver lesions (57.2%). Early washout within ≤60 seconds was observed in 32 lesions, among which nine lesions (28.1%) were HCC, one lesion (3.1%) was an inflammatory pseudo-tumour, and 22 lesions (68.8%) were other malignancies (including lymphoma, intrahepatic cholangiocarcinoma [ICC], and HCC-ICC) on histopathological analysis. Twenty lesions showed marked washout within 120 seconds, including two HCCs and 18 ICCs (Figs. 3, 4 and 5).

### Diagnostic accuracy in the retrospective study

Table 3 shows the frequency of the CEUS LI-RADS categories in the RF+ and RF− groups and the incidence of HCC and malignant tumours in each category. No malignant lesions with CEUS LR-1 and LR-2 categories were observed in either group. The incidence of HCC based on the LR-3 classification was 12.0% (3/25) in the RF+ group and 14.3% (3/21) in the RF− group. The incidence of HCC based on the LR-4 classification was 58.8% (30/51) in the RF+ group and 32.7% (32/98) in the RF− group (*P* = 0.002). The incidence of benign tumours based on the LR-4 classification was 41.2% (21/51) in the RF+ group and 60.2% (59/98) in the RF− group (*P* = 0.027).

**Table 3:** Nodules in CEUS LI-RADS categories in retrospective study

Category	RF+				RF−			
	No. of Nodules (*n* = 328)^a^	Incidence of HCC %	Incidence of Malignancy %	Incidence of Benign%	No. of Nodules (*n* = 418)^a^	Incidence of HCC %	Incidence of Malignancy %	Incidence of Benign%
CEUS LI-RADS								
LR-1	48 (14.6)	0	0	100 (48/48)	71 (17.0)	0	0	100 (71/71)
LR-2	0	0	0	0	1 (0.2)	0	0	100 (1/1)
LR-3	25 (7.6)	12 (3/25)	12 (3/25)	88 (22/25)	21 (5.0)	14 (3/21)	14 (3/21)	86 (18/21)
LR-4	51 (15.5)	59 (30/51)	59 (30/51)	41 (21/51)	98 (23.4)	33 (32/98)	33 (32/98)	67 (59/98)
LR-5	169 (51.5)	96 (162/169)	96 (163/169)	4 (6/169)	176 (42.1)	90 (158/176)	91 (160/176)	9 (16/176)
LR-M	35 (10.6)	31 (11/35)	97 (34/35)	3 (1/35)	51 (12.2)	22 (11/51)	98 (50/51)	2 (1/51)

*Note*: Unless otherwise indicated, data in parentheses are numerators/denominacors. *CEUS LI-RADS* contrast-enhanced US Liver Imaging Reporting and Data System, *HCC* hepatocellular carcinoma

^a^Data in parentheses are percentages

The positive predictive value (PPV) of CEUS LR-5 for HCC lesions was significantly higher in the RF+ group than in the RF− group (*P* = 0.029). However, the sensitivities and specificities did not differ between the RF+ and RF− groups (*P* = 0.771 and *P* = 0.369, respectively; Table 4).

**Table 4:** Diagnostic Performance of CEUS LI-RADS in RF+ and RF− patients in Retrospective Study

Criteria	Sensitivity	Specificity	Accuracy	PPV	NPV
CEUS LR-5 in RF+ group	78.6 (162/206) [72.4,84.0]	94.3 (115/122) [88.6,96.7]	84.5 (277/328) [76.8,85.5]	95.9 (162/169) [85.9,98.7]	72.3 (115/159) [64.5,75.8]
CEUS LR-5 in RF− group	77.5 (158/204) [71.1,83.0]	91.6 (196/214) [88.5,98.7]	84.7 (354/418) [76.8,87.5]	89.8 (158/176) [87.2,96.5]	81.0 (196/242) [75.1,83.6]
*P*	0.771	0.369	0.929	0.029	0.042

*Note*: Data in parentheses are numerator/denominator and data in brackets are 95% confidence intervals. *CEUS LI-RADS* conrrast-enhanced US Liver Imaging Reporting and Data System, *PPV* Positive predictive value, *NPV* Negative predictive value

### Diagnostic accuracy validation in a prospective real-life setting

The prospective study included 38 lesions from 36 patients (mean age, 47.8 ± 11.6 years; males, 25 [65.8%]) in the RF− group (Fig. 1); two patients had two lesions each. The average nodule size was 31.8 ± 12.7 (9–167) mm. Eighteen (47.4%) lesions were HCC. The RF+ group included 89 lesions from 84 patients, with two lesions each in five patients; the mean lesion diameter was 34.3 ± 17.6 (11–171) mm. Fifty-five (62.8%) lesions were HCC. The time interval between the CEUS and surgery was 7 ± 4 days. All histopathologic tissue analysis of the lesions was conducted using surgical specimens.

In the prospective study, the sensitivity, specificity, PPV, negative predictive value (NPV), and diagnostic accuracy of CEUS LR-5 in the RF− group were 72.2% (13/18), 90.0% (18/20), 86.7% (13/15), 78.2% (18/23), and 81.5% (31/38), respectively. The sensitivity, specificity, PPV, NPV, and diagnostic accuracy of CEUS LR-5 in the RF+ group were 74.5% (41/55), 94.1% (32/34), 95.3% (41/43), 69.6% (32/46), and 82.0% (73/89), respectively. The PPV of CEUS LR-5 for HCC lesions was significantly higher in the RF+ group than in the RF− group (*P* = 0.030). The sensitivities and specificities did not differ between the RF+ and RF− groups in the prospective study (*P* = 0.845 and *P* = 0.577, respectively; Table 5).

**Table 5:** Diagnostic Performance of CEUS LI-RADS in RF+ and RF− patients in prospective study

Criteria	Sensitivity (%)	Specificity (%)	Accuracy (%)	PPV	NPV
CEUS LR-5 in RF+ group	74.5 (41/55) [70.1,84.8]	94.1 (32/34) [86.1,97.7]	82.0 (73/89) [75.8,88.5]	95.3 (41/43) [88.9,97.6]	69.6 (32/46) [58.5,76.1]
CEUS LR-5 in RF− group	72.2 (13/18) [65.3,78.2]	90.0 (18/20) [83.4,94.5]	81.5 (31/38) [74.3,88.1]	86.7 (13/15) [81.2,92.3]	78.2 (18/23) [72.1,85.4]
*P*	0.845	0.577	0.953	0.030	0.446

*Note*: Data in parentheses are numerator/denominator and data in brackets are 95% confidence intervals. *CEUS LI-RADS* conrrast-enhanced US Liver Imaging Reporting and Data System, *PPV* Positive predictive value, *NPV* Negative predictive value

## Discussion

The CEUS LI-RADS provides tools to standardise imaging diagnoses in patients with liver tumours. Previous LI-RADS studies on patients at risk of HCC have mainly focused on those with HBV infections [4, 5]. Consequently, the diagnostic performance of the LI-RADS in RF− patients has not been fully evaluated, and there is an urgent need to study the diagnostic performance of this system in the RF− population. Our research showed that the sensitivities, specificities, and diagnostic coincidence rates were similar between the RF− and RF+ groups in the CEUS LR-5 category. However, the CEUS LR-5 PPV was higher in the RF+ group than in the RF− group.

Terzi et al. reported that the PPV of CEUS LR-5 in RF+ patients was 98.5% [5], and Huang et al. reported a PPV of 97.5% [4]. In our study, the PPVs of CEUS LR-5 for HCC in the RF+ group were 95.9% (162/169) and 95.35% (41/43) in the retrospective and prospective studies, respectively, similar to the values reported by Terzi et al. and Huang et al. Furthermore, the PPVs of CEUS LR-5 for HCC in the RF− group were 89.8% (158/176) and 86.7% (13/15) in the retrospective and prospective studies, respectively, lower than the values reported by Terzi et al. and Huang et al. This result implies that benign lesions incorrectly classified as LR-5 were far more common in the RF- group than in the RF+ group. In our retrospective study, the PPVs of CEUS LR-M were 97% (34/35) and 98% (50/51) in the RF+ and RF– groups, respectively (*P* = 0.786). The PPVs of the CEUS LR-M category in the RF+ and RF− groups were comparable in the prospective study.

The LR-4 category was designed to classify lesions that were probably but not definitively HCC. Terzi et al. reported that 85.6% of HCC lesions were categorised as LR-4 in the RF+ group [5]. In contrast, Huang et al. [4] reported that 48% of HCC lesions were categorised as LR-4 in the RF+ group. However, in our retrospective study, only 32.7% (32/98) of HCC lesions were categorised as LR-4 in the RF− group. Furthermore, these benign lesions categorised as LR-4 in the RF− group included eight haemangiomas, two focal nodular hyperplasias, four biliary adenomas, ten neuroendocrine neoplasms, six angiomyolipomas, 18 dysplastic lesions, two inflammatory pseudo-tumours, and six lipomas. Also, the contrast agent appeared to be significantly enhanced after entering the rich vascular network of the haemangioma, focal nodular hyperplasia, inflammatory pseudo-tumour, or angiomyolipoma. The haemodynamics of the contrast agents may overlap between high-grade dysplastic lesions and well-differentiated HCC [19]. Therefore, in RF− patients, radiologists should be more cautious regarding the diagnoses of patients classified as CEUS LR-4 and thoroughly consider the necessity of a needle biopsy. However, clinicians should neither miss the HCC diagnosis nor try to avoid invasive examinations of patients with benign lesions. Thus, clinicians may be required to make a comprehensive judgement in conjunction with laboratory examinations.

In this study, the NPV differed between the retrospective and prospective sets; it was significantly lower in the RF+ group than RF– group in the retrospective setting, whereas there was no difference in the prospective setting. The proportion of non-HCC/non-LR-5 in the RF– group was greater because the proportion of non-HCC patients in the RF– group (51.2% [214/418]) was greater than that in the RF+ group (37.2% [122/328]). Unlike retrospective studies, where the reference standard consists of histologic evaluation or composite imaging and clinical follow-up findings, prospective studies use surgical pathology results as the gold standard since they are the most accurate and reliable. Retrospective studies and prospective validation sets have a selection bias for benign lesions. In contrast, all malignant lesions, including HCC and non-HCC malignancies, were diagnosed based on histopathologic examination findings; thus, selection bias does not exist for malignant lesions.

This study has some limitations. First, our data were obtained exclusively from Chinese patients, and the distribution of clinical characteristics of patients in other geographic regions may be different. Furthermore, this study was a single-centre study, which limits its scope. Future prospective studies should recruit more patients from multiple centres to study the diagnostic value of the CEUS LI-RADS in RF− patients. Finally, the number of small lesions (< 10 mm) was relatively small, limiting the diagnostic value for small lesions. These issues require further study.

## Conclusion

In patients with and without HCC associated risk factors, the CEUS LR-5 criteria also shows clinical value for diagnosis of HCC. And patients categorised as CEUS LR-4 in the RF− group require a more careful and comprehensive consideration by clinicians.

## Data Availability

The dataset used or analyzed during the current study are available from the corresponding author on reasonable request.

## Abbreviations

HCC: Hepatocellular carcinoma
CEUS: Contrast-Enhanced Ultrasound
LI-RADS: Liver Imaging Reporting and Data System
RF−: Without LI-RADS-defined HCC risk factors
RF+: With LI-RADS-defined HCC risk factors
PPV: Positive predictive value
LR-1: LI-RADS category 1
HBV: Hepatitis B virus
NAFLD: non-alcoholic fatty liver disease
NPV: Negative predictive value
